# Standardized Treatment of Active Tuberculosis in Patients with Previous Treatment and/or with Mono-resistance to Isoniazid: A Systematic Review and Meta-analysis

**DOI:** 10.1371/journal.pmed.1000150

**Published:** 2009-09-15

**Authors:** Dick Menzies, Andrea Benedetti, Anita Paydar, Sarah Royce, Madhukar Pai, William Burman, Andrew Vernon, Christian Lienhardt

**Affiliations:** 1 Respiratory and Epidemiology Clinical Research Unit, Montreal Chest Institute, McGill University, Montreal, Canada; 2 University of California at San Francisco, San Francisco, California, United States of America; 3 Denver Public Health, Denver, Colorado, United States of America; 4 Centers for Disease Control and Prevention, Atlanta, Georgia, United States of America; 5 International Union against Tuberculosis and Lung Diseases, and Institut de Recherche pour le Développement, Paris, France; Harvard School of Public Health, United States of America

## Abstract

**Background:**

A standardized regimen recommended by the World Health Organization for retreatment of active tuberculosis (TB) is widely used, but treatment outcomes are suspected to be poor. We conducted a systematic review of published evidence of treatment of patients with a history of previous treatment or documented isoniazid mono-resistance.

**Methods and Findings:**

PubMed, EMBASE, and the Cochrane Central database for clinical trials were searched for randomized trials in previously treated patients and/or those with with mono-resistance to isoniazid, published in English, French, or Spanish between 1965 and June 2008. The first two sources were also searched for cohort studies evaluating specifically the current retreatment regimen. In studies selected for inclusion, rifampin-containing regimens were used to treat patients with bacteriologically confirmed pulmonary TB, in whom bacteriologically confirmed failure and/or relapse had been reported. Pooled cumulative incidences and 95% CIs of treatment outcomes were computed with random effects meta-analyses and negative binomial regression. No randomized trials of the currently recommended retreatment regimen were identified. Only six cohort studies were identified, in which failure rates were 18%–44% in those with isoniazid resistance. In nine trials, using very different regimens in previously treated patients with mono-resistance to isoniazid, the combined failure and relapse rates ranged from 0% to over 75%. From pooled analysis of 33 trials in 1,907 patients with mono-resistance to isoniazid, lower failure, relapse, and acquired drug resistance rates were associated with longer duration of rifampin, use of streptomycin, daily therapy initially, and treatment with a greater number of effective drugs.

**Conclusions:**

There are few published studies to support use of the current standardized retreatment regimen. Randomized trials of treatment of persons with isoniazid mono-resistance and/or a history of previous TB treatment are urgently needed.

*Please see later in the article for the Editors' Summary*

## Abstract 2

Performing a systematic review of studies evaluating retreatment of tuberculosis or treatment of isoniazid mono-resistant infection, Dick Menzies and colleagues find a paucity of evidence to support the WHO-recommended regimen.

## Editors' Summary

**Background:**

Every year, nearly ten million people develop tuberculosis—a contagious infection, usually of the lungs—and about 2 million people die from the disease. Tuberculosis is caused by *Mycobacterium tuberculosis*, bacteria that are spread in airborne droplets when people with the disease cough or sneeze. Its symptoms include a persistent cough, fever, weight loss, and night sweats. Diagnostic tests for tuberculosis include chest X-rays and sputum slide exams and cultures in which bacteriologists try to grow *M. tuberculosis* from mucus brought up from the lungs by coughing. The disease can be cured by taking several powerful antibiotics regularly (daily or several times a week) for at least 6 months. However, 10%–20% of patients treated for tuberculosis in low- and middle-income countries need re-treatment because the initial treatment fails to clear *M. tuberculosis* from their body or because their disease returns after they have apparently been cured (treatment relapse). Patients who need re-treatment are often infected with bacteria that are resistant to one or more of the antibiotics commonly used to treat tuberculosis.

**Why Was This Study Done?:**

As part of its strategy to reduce the global burden of tuberculosis, the World Health Organization (WHO) recommends standardized treatment regimens for tuberculosis. For re-treatment, WHO recommends an 8-month course of isoniazid, rifampin, and ethambutol with pyrazinamide and streptomycin added for the first 3 and 2 months, respectively. All these drugs are given daily (the preferred regimen) or three times a week. Unfortunately, although this regimen is now used to treat about 1 million patients each year, it yields poor results, particularly in regions where drug resistance is common. In this study (which was commissioned by WHO to provide the evidence needed for a revision of its treatment guidelines), the researchers undertake a systematic review (a search using specific criteria to identify relevant research studies, which are then appraised) and a meta-analysis (a statistical approach that pools the results of several studies) of randomized trials and cohort studies (two types of study that investigate the efficacy of medical interventions) of re-treatment regimens in previously treated tuberculosis patients, and in patients with infection that was resistant to isoniazid (“mono-resistance”).

**What Did the Researchers Do and Find?:**

The researchers' systematic search for published reports of randomized trials and cohort studies of the currently recommended re-treatment regimen identified no relevant randomized trials and only six cohort studies. In the three cohort studies in which the participants carried *M. tuberculosis* strains that were sensitive to all the antibiotics in the regimen, failure rates were generally low. However, in the studies in which the participants carried drug-resistant bacteria, failure rates ranged from 9% to 45%. The researchers also identified and analyzed the results of nine trials in which several re-treatment regimens, all of which deviated from the standardized regimen, were used in previously treated patients with isoniazid mono-resistance. In these trials, the combined failure and relapse rates ranged from 0% to more than 75%. Finally, the researchers analyzed the pooled results of 33 trials that investigated the effect of various regimens on nearly 2,000 patients (some receiving their first treatment for tuberculosis, some being re-treated) with isoniazid mono-resistance. This meta-analysis showed that lower relapse, failure, and acquired drug resistance rates were associated with longer duration of rifampicin treatment, use of streptomycin, daily therapy early in the treatment, and regimens that included a greater number of drugs to which the *M. tuberculosis* carried by the patient were sensitive.

**What Do These Findings Mean?:**

These findings reveal that there is very little published evidence that supports the regimen currently recommended by WHO for the re-treatment of tuberculosis. Furthermore, this limited body of evidence is a patchwork of results gleaned from a few cohort studies and a set of randomized trials not specifically designed to test the efficacy of the standardized regimen. There is an urgent need, therefore, for a concerted international effort to initiate randomized trials of potential treatment regimens in both previously untreated and previously treated patients with all forms of drug-resistant tuberculosis. Because these trials will take some time to complete, the limited findings of the meta-analysis presented here may be used in the meantime to redesign and, hopefully, improve the current standardized re-treatment regimen. In fact, the revised WHO TB treatment guidelines will provide updated recommendations for patients with previously treated TB.

**Additional Information:**

Please access these Web sites via the online version of this summary at http://dx.doi.org/10.1371/journal.pmed.1000150.

- The results of another WHO-commissioned study into the treatment of tuberculosis are presented in a separate *PLoS Medicine* Research Article by Menzies et al. (Menzies D, Benedetti A, Paydar A, Martin I, Royce S, et al. (2009) Effect of Duration and Intermittency of Rifampin on Tuberculosis Treatment Outcomes: A Systematic Review and Meta-Analysis. PLoS Med 6(9): e1000146.)
- The US National Institute of Allergy and Infectious Diseases provides information on all aspects of tuberculosis
- The American Thoracic Society, US Centers for Disease Control and Prevention, and Infectious Diseases Society of America offer guidelines on TB treatment
- The US Centers for Disease Control and Prevention provide several facts sheets and other information resources about tuberculosis
- The 2003 (2004 revision) WHO guidelines for national programs for the treatment of tuberculosis are available; WHO also provides information on efforts to reduce the global burden of tuberculosis (in several languages) and its 2009 annual report on global control of tuberculosis describes the current situation (key points are available in several languages)
- The WHO publishes guidelines on TB treatment
- For guidelines on drug susceptibility testing (DST) and other information on TB diagnostic tests, the Stop TB Partnership's New Diagnostics Working Group has created a new Web site called Evidence-Based Tuberculosis Diagnosis

## Introduction

A key component of the directly observed treatment short-course (DOTS) strategy of the World Health Organization (WHO) is the use of a limited number of standardized regimens [1]. This strategy ensures that treatment is always given with the same number, dose, and type of medications—the simplicity enhances access to treatment in resource-poor settings. A single 8-mo “retreatment” regimen (8 mo of isoniazid, rifampin, ethambutol, with pyrazinamide added for the first 3 mo, and streptomycin added for the first 2 mo—2SHRZE/1HRZE/5HRE) is recommended for all patients with a history of previous treatment [1] and is used in more than 90 countries [2].

This WHO retreatment regimen was initially designed for resource-poor settings with low prevalence of initial drug resistance, and for patients previously treated with a regimen that used rifampin only for the first 2 mo of therapy [3]. It was believed that the regimen should be adequate for patients with mono-resistance, such as to isoniazid. However, this regimen has been increasingly criticized [4],[5] because of poor results [2], particularly in settings where prevalence of initial drug resistance is high [2],[6] or rifampin is used throughout initial therapy [7]. In light of these concerns, we have conducted a systematic review of published evidence of treatment of patients with a history of previous treatment or documented isoniazid mono-resistance.

## Review Questions

Our systematic review addressed two specific questions:

1. What are the rates of failure, relapse, and acquired drug resistance with the currently recommended WHO retreatment regimen—in randomized trials and cohort studies?
2. What treatment factors are associated with failure, relapse, and acquired drug resistance in randomized trials of patients with pretreatment resistance to isoniazid?

## Methods

### Search Strategy

**Figure 1:**
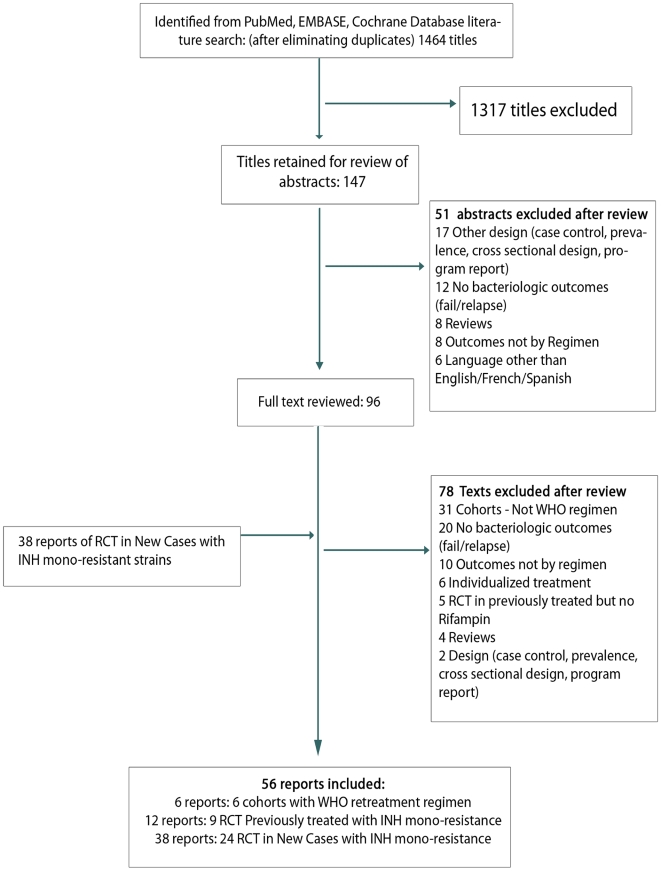
Summary of study review and selection.

As seen in Figure 1, we searched three electronic databases—PubMed, EMBASE, and the Cochrane Central database—for randomized controlled trials (RCTs) of treatment of patients with previous TB treatment, or mono-resistance to isoniazid. The search was restricted to studies published in English, French, or Spanish between 1965 and June 2008. Our keywords included tuberculosis or TB, retreatment or repeated therapy or previously treated patients, and failure or relapse or drug resistance. To identify additional relevant articles, we searched reference lists of identified original articles, recent reviews [8], chapters from texts [9],[10], and recent treatment guidelines [1],[11],[12].

### Study Selection

In selected studies, all patients had bacteriologically confirmed (by smear and/or culture) active pulmonary TB, and reported treatment outcomes of bacteriologically confirmed failure and/or relapse. Treatment was standardized and included at least rifampin. We excluded studies or arms that utilized rifapentine, rifabutin, or nondrug therapy (for example, immunotherapeutic vaccines). We also excluded regimens with once weekly or single drug therapy, as these would now be considered inadequate [11]–[13].

The selection of articles for review was done independently by two investigators in three stages: titles alone, then abstracts, and then full text articles. Decisions were compared and disagreements about study selection were resolved by consensus or by involving a third reviewer. We initially planned to select only RCTs in previously treated patients. We could not identify any randomized trial that evaluated specifically the current WHO retreatment regimen (2SHRZE/1HRZE/5HRE). Therefore, we included any cohort studies that used this specific regimen, reported individual outcomes, and met all inclusion criteria above.

We found only 11 randomized trials in previously treated patients, of which nine evaluated patients with isoniazid mono-resistance (these trials used several different regimens, but all different from the WHO retreatment regimen). Therefore, we decided to include trials, identified in a separate systematic review, in which previously untreated patients with mono-resistance to isoniazid were treated with standardized rifampin containing regimens. (For methods, see Menzies et al. [14].)

### Data Extraction and Quality Assessment

We used standardized forms to extract data from selected studies about patient population and characteristics, treatment regimen, and pretreatment drug-susceptibility testing—if done, supervision of treatment, funding source, and number of patients who started treatment, died, failed, relapsed, defaulted, or were otherwise lost. Two reviewers extracted the data, with disagreements resolved by consensus.

The selection criteria requiring microbiologic confirmation of initial diagnosis and treatment outcomes meant that selected studies had high-quality diagnostic methods. Another indicator of study quality was the number of patients who refused therapy, dropped out, moved away, or were otherwise unaccounted for during therapy. Trials were considered to have high-quality methods of randomization if they used central randomization, numbered opaque sealed envelopes, sealed envelopes from a closed bag, or numbered or coded bottles or containers.

### Outcomes

Treatment failure was defined as sputum smears and/or cultures that were consistently positive or positive requiring treatment after at least 5 mo of therapy [15]. Relapse was defined as recurrence of positive smears and/or cultures that required therapy 1 mo or more after apparent cure [15]. Acquired drug resistance was defined as new or additional resistance to one or more of the TB drugs received—in the setting of failure or relapse. Patients with pretreatment rifampin resistance, including multidrug resistance, were excluded from analysis, if identified in the published report.

### Data Synthesis and Analysis

We were interested to understand the efficacy of different regimens in preventing failure, relapse, and acquired drug resistance—end-points with objective microbiological definitions that were consistent across trials. Therefore, we used a per-protocol analysis, excluding patients who did not complete therapy because they developed serious adverse reactions, died, defaulted, dropped out, or were otherwise not accounted for.

Only 6 cohort studies were found that evaluated the current WHO retreatment regimen; these results were not pooled. Of 11 trials in previously treated patients, nine included patients with mono-resistance to isoniazid, one included patients only with poly-drug resistant strains [16], while another, which included patients with multiple forms of resistance, did not report results by type of drug resistance [17]. Results from the last two trials were not analyzed. To increase power, results from the nine trials in isoniazid mono-resistant previously treated patients were pooled with results of the subgroups of patients with mono-isoniazid resistance in 24 trials in new cases.

Very few trials with head-to-head comparisons were identified, and no two trials made the same comparisons. Hence we pooled results across all trials, effectively treating each arm within each trial as an independent cohort. For this across-trial analysis, we used a random effects meta-analysis to estimate the overall pooled estimates of cumulative incidence and 95% CI of failure, relapse, and acquired drug resistance using PROC NLMIXED in SAS (SAS institute, Carey, NC, USA) [18]. We used the exact binomial likelihood approach [18], which uses a binomial distribution to approximate the distribution of the outcome of interest. This approach accounts for study size and includes a random effect to account for inter-study heterogeneity. When proportions are the outcome measure, this approach has been demonstrated to produce less-biased estimates of the pooled effect and the between-study variability [18]. To minimize heterogeneity, we performed subgroup analyses stratified by predefined covariates of interest. These included the following characteristics of the re-treatment regimen duration and dosing schedule of rifampin, initial drug resistance, use of pyrazinamide or streptomycin, and number of drugs to which the organism was susceptible used in the initial or continuation phase (the initial intensive phase was defined as the initial period when more drugs were used—usually the first 1–2 mo—while the continuation phase was the remainder of therapy). We also examined the effect of supervision of therapy, proportion that were smear positive, and default or other losses during treatment phase follow-up. We assessed heterogeneity of outcomes of interest, within subgroups defined by covariates of interest by estimating the I^2^ statistic and associated 95% CIs [19].

Finally, meta-regression was used to estimate the effect of the treatment factors of interest, after adjustment for other potentially confounding patient and treatment covariates. Because the outcomes we were pooling were proportions, rather than odds ratios, and because these proportions were usually small, we performed meta-regression using the Poisson model [20] that allowed for overdispersion (i.e., negative binomial regression). In this meta-regression, each arm in each study was the unit of analysis, cumulative incidence of TB treatment outcomes was the dependent variable, and TB treatment characteristics were the independent variables. An offset was used to account for size of study. In this approach, residual heterogeneity between studies is accounted for in the dispersion parameter. As such, we interpreted the dispersion parameter as indicating there was no remaining unexplained heterogeneity if the value was not significantly different from 0, and as minimal heterogeneity if the value was less than 1.0 [21]. Effect estimates of the meta-regression model were interpreted as adjusted incidence rate ratios [20]. We tested the significance of each factor in the models using the likelihood ratio test.

The final model included rifampin duration, intermittent schedule, use of pyrazinamide and streptomycin, number of drugs in initial or continuation phases to which organisms were susceptible, length of follow-up after end of treatment (for relapse and acquired drug resistance), supervision of therapy (directly observed therapy [DOT]), and noncompletion of therapy because of protocol violations, patient refusal, default, moved, or lost.

## Results

### Study Selection

As seen in Figure 1, 1,464 citations were identified from the search of the three electronic databases. Of these, 147 were retained for abstract review and 96 for full text review. In total, six cohort studies were found describing results with the standardized 8-mo retreatment in previously treated patients with pretreatment drug susceptibility testing (DST) [6],[22]–[26], and 12 reports describing nine randomized trials in previously treated patients with isoniazid mono-resistance [27]–[38]. To these, we added results in previously untreated patients with initial mono-resistance to isoniazid taken from 38 published reports describing 24 randomized trials [39]–[75]. The initial diagnosis and outcomes were confirmed with cultures (i.e., not just smears) in all randomized trials. In the trials, rifampin dose was 600 mg (or 10–12 mg/kg) in all trials except three trials that used 450 mg daily [29],[33],[67], one that used 450 mg daily followed by 600 mg thrice weekly [34], and one with 450 mg daily followed by 900 mg twice weekly [31]. Other characteristics of the studies and study populations are summarized in Table S1.

### Results from Cohort Studies with Standardized WHO Retreatment Regimen

As shown in Table 1, failure rates were low in most cohorts with pan-sensitive strains that received the standardized retreatment regimen, although the rate was inexplicably high in one study [25]. In cohorts with mono-resistance to isoniazid, failure rates ranged from 18%–44%, compared to 9%–45% in cohorts whose drug resistance patterns were not reported. Pooled estimates were not calculated because of heterogeneity in results.

**Table 1:** Cohort studies reporting results with standardized retreatment regimen recommended by WHO.

ID	Reference	Regimen^a^	Total Number Treated	Number at Risk for Failure^b^	Number (%) Who Failed	Number at Risk for Acquired Drug Resistance^c^	Number with Acquired Drug Resistance
**Pan-sensitive strains**							
33	[22]	2HRZES/1HRZE/5HRE	382	306	2 (0.7%)	306	1
34	[23]	2HRZES/1HRZE/5HRE	30	28	0	28	0
340	[25]	2HRZES/1HRZE/5HRE	122	87	5 (6%)	—	—
		2HRZES/1HRZE/5[HRE]_2_	260	208	13 (6%)	—	—
		2HRZES/1HRZE/5[HRE]_3_	104	64	17 (27%)	—	—
**Mono-resistance to INH**							
340	[25]	2HRZES/1HRZE/5HRE	57	39	7 (18%)	—	—
		2HRZES/1HRZE/5[HRE]_2_	37	31	6 (19%)	—	—
		2HRZES/1HRZE/5[HRE]_3_	30	18	8 (44%)	—	—
**Mixed drug resistance (all forms or unknown)**							
324	[24]	2[HRZES]_3_/1[HRZE]_3_/5[HRE]_3_	57	46	4 (9%)	—	—
		2[HRZES]_3_/2[HRZE]_3_/5[HRE]_3_	17	11	5 45%)	—	—
384	[6]	2HRZES/1HRZE/5EHR	210	183	47 (26%)	—	—
415	[26]	2[HRZES]_3_/1[HRZE]_3_/5[HRE]_3_	507	389	52 (13%)	—	—

None of these studies ascertained relapse after treatment completion.

a Regimen abbreviations: H, isoniazid; R, rifampin; Z, pyrazinamide; E, ethambutol; S, streptomycin. Letters to left of “/” indicate regimen in initial intensive phase; letters to right of “/” indicate regimen in continuation phase. First number signifies the months of initial phase of treatment and the second number signifies the months of continuation phase treatment. [ ] indicate intermittent therapy; subscript number after [ ] indicates number of doses per week.

b Number at risk for failure excluded those who died, defaulted, or had serious adverse reactions during therapy (i.e., included only those who completed treatment or failed).

c If acquired drug resistance was not measured, this is indicated as “—”.

### Results from RCTs in Previously Treated Patients with INH Mono-resistance

In the nine randomized trials, summarized in Table 2, randomization was high quality in eight, and treatment phase losses were less than 10% in six. Regimens and treatment outcomes varied widely. In three studies that used the standard therapy for drug-sensitive TB of 2HRZ (±E)/4HR [28],[29],[32], the combined failure and relapse rates ranged from 29%–70%. In four studies [30],[31],[34]–[38] in which patients were given rifampin plus ethambutol for 12 mo or more, failure rates ranged from 4%–23% and relapse rates from 0%–27%. The regimen most like the current retreatment regimen was evaluated in a single trial [27]. A total of 179 evaluable patients received 2SRZE/4RE or 2SRZE/7RE with failure and relapse rates of 1% and 3%, respectively [27]. In one arm of one trial, among 89 patients who received a similar regimen of 3REZ/9RE (albeit thrice weekly), failure and relapse rates were 23% and 20%, respectively [34].

**Table 2:** Regimens and results in randomized trials in previously treated patients with INH mono-resistance.

ID	Reference	Regimen^a^	Total Number Treated	At Risk for Failure (*N*)^b^	Number (%) who Failed	At Risk for Relapse (*N*)^c^	Number (%) who Relapsed	At Risk for Acquired Drug Resistance (*N*)^b^	Number (%) with Acquired Drug Resistance
14	[27]	2SZRE/4RE	101	91	0	72	3 (4%)	91	0
		2SZRE/7RE	97	88	1 (1%)	72	2 (3%)	88	0
23	[28]	2[HRZE]_3_/4[HR]_2_	171	167	32 (19%)	135	14 (10%)	167	23 (14%)
50	[29]	2HRZE/6EH	101	94	16 (17%)	73	6 (8%)	94	3 (3%)
		2[HRZE]_2_/4[HRE]_2_	63	59	12 (20%)	44	11 (25%)	59	7 (12%)
		2[HRZ]_2_/4[HR]_2_	79	74	46 (62%)	27	4 (15%)	74	13 (18%)
302	[34]	**Subgroup with isoniazid mono-resistance**							
		12RE *(RIF 450)*	83	73	20 (27%)	19	5 (26%)	73	19 (26%)
		12[RE]_3_ *(RIF 450)*	91	75	22 (29%)	22	6 (27%)	75	21 (28%)
		3[REZ]_3_/9[RE]_3_ *(RIF 450)*	89	77	18 (23%)	30	6 (20%)	77	17 (22%)
		3[RE]_3_/9[RE]_3_ *(RIF 450)*	94	75	10 (13%)	35	12 (34%)	75	10 (13%)
302	[34]	**Subgroup with isoniazid and ethambutol resistance**							
		12RE *(RIF 450)*	25	15	10 (67%)	5	1 (20%)	—	—
		12[RE]_3_ *(RIF 450)*	22	12	8 (67%)	4	1 (25%)	—	—
		3[REZ]_3_/9[RE]_3_ *(RIF 450)*	20	12	5 (42%)	7	0	—	—
		3[REPt]_3_/9[RE]_3_ *(RIF 450)*	16	6	2 (33%)	4	1 (25%)	—	—
326	[31]	12ER *(RIF 450)*	112	106	11 (10%)	—	—	106	9 (8%)
		12[ER]_2_ *(RIF 450)*	93	87	16 (18%)	—	—	87	15 (17%)
328	[32]	2HRZ/4HR	9	9	3 (33%)	6	1 (17%)	6	0
		2HRZ/4(HR)_2_	4	4	1 (25%)	3	0	3	0
357	[33]	6HRZ	9	9	0	—	—	—	—
		6HRE	10	10	5 (50%)	—	—	—	—
		6HRZ	15	9	0	—	—	—	—
		6HRE	15	10	5 (50%)	—	—	—	—
400	[30],[35],[36],[38]	3RE/9[RE]_2_	43	40	2 (5%)	38	2 (5%)	—	—
		3RE/9[RE]_2_ *(RIF 1200)*	42	39	2 (5%)	37	0	—	—
		3RE/15[RE]_2_ *(RIF 1200)*	43	40	1 (3%)	40	2 (5%)	—	—
		3RE/21[RE]_2_ *(RIF 1200)*	42	39	1 (3%)	38	0	—	—
416	[37]	1.5RE/10.5[RE]_3_	34	30	2 (7%)	23	0	—	—
		12[RE]_3_	38	33	4 (12%)	25	0	—	—

Dose of rifampin = 600 mg daily unless indicated otherwise.

a Regimen abbreviations: H, isoniazid; R, rifampin; Z, pyrazinamide; E, ethambutol; S, streptomycin. Letters to left of “/” indicate regimen in initial intensive phase; letters to right of “/” indicate regimen in continuation phase. First number signifies the months of initial phase of treatment and the second number signifies the months of continuation phase treatment. [ ] indicate intermittent therapy; subscript number after [ ] indicates number of doses per week.

b Number at risk for failure, relapse, or acquired drug resistance excluded those who died, defaulted, or had serious adverse reactions during therapy (i.e., included only those who completed treatment or failed).

c If relapse or acquired drug resistance not measured, it is indicated as “—”.

### Pooled Results for Isoniazid Mono-resistance from All Trials

In the 24 randomized trials in new cases, randomization was high quality in 16, but not specified in eight, and treatment phase losses were less than 10% in 18 (67%). In the total of 33 trials with patients with isoniazid mono-resistance, treatment failure was assessed in 1,907 patients within 101 different treatment arms, relapse in 1,196 persons in 93 arms, and acquired drug resistance was assessed in 1,484 patients in 76 different arms. As seen in Table 3, failure was significantly lower if streptomycin was used, or if a larger number of effective drugs were used in the initial or continuation phases. Failure rates progressively fell with longer use of pyrazinamide, although all CIs overlapped. There was also a trend for higher failure rates if rifampin was used for only 2 mo, or drugs were administered biweekly throughout therapy. Results were very similar when the analysis was restricted to high-quality studies—in which less than 10% of participants dropped out, moved, or were otherwise lost to follow-up during the treatment phase.

**Table 3:** Stratified analysis of covariates associated with TB treatment outcomes in RCT of patients with INH resistance in new or previously treated cases: Failure in isoniazid mono-resistance.

Factor	Arms (*N*)	Events/Patients (*N*)	Pooled Event Rate	95% CI	I^2^ (95% CI)
**Rifampin use**					
Rifampin 1–2 mo	19	30/256	6.2	0–12.8	0 (0–0.48)
Rifampin 3–5 mo	10	2/88	0.9	0–2.9	0 (0–0.60)
Rifampin 6–7 mo	46	108/645	4.8	0.8–8.8	0.76 (0.69–0.82)
Rifampin 8+ mo	19	136/858	7.4	0–15.1	0.87 (0.82–0.91)
**Frequency of therapy in the initial intensive phase** a					
Daily	65	99/1,062	5.1	2.2–8.0	0.50 (0.33–0.62)
Thrice weekly	25	102/559	5.2	0–10.5	0.44 (0.10–0.65)
Twice weekly	4	75/226	25.5	0–52.8	0.93 (0.86–0.97)
**Duration of PZA**					
No PZA	28	123/769	**11.4**	**4.0–18.8**	0.74 (0.63, 0.82)
1–3 mo	39	147/871	6.6	2.3–10.9	0.83 (0.77–0.87)
4 mo or more	27	6/207	1.7	0–3.6	0 (0–0.42)
**Duration of streptomycin**					
No streptomycin	43	258/1,294	**13.6**	**7.8–19.3**	0.80 (0.73–0.85)
1–3 mo	28	13/383	2.8	0.6–5.0	0 (0–0.41)
4 mo or more	23	5/170	2.1	0–4.5	0 (0–0.46)
**Number of drugs to which strains susceptible (effective drugs)**					
***Initial phase*** b					
1 drugs	3	22/39	**50.8**	**6.1–9.6**	0.52 (0–0.86)
2 drugs	31	99/628	11.3	0.3–19	0.58 (0.37–0.72)
3 drugs	55	148/932	3.8	0.9–6.7	0.58 (0.43–0.69)
4 or more drugs	3	1/185	0.4	0–1.5	0 (0–0.73)
***Continuation phase***					
0–1 drugs	44	153/688	**9.1**	**3.0–15.2**	0.71 (0.61–0.79)
2 drugs	35	116/998	**6.0**	**1.6–10.5**	0.72 (0.61–0.80)
3 or more drugs	11	1/92	0.5	0–1.6	0 (0–0.58)
**Supervision of therapy**					
All doses fully supervised	69	148/1,341	3.8	1.2–6.3	0.59 (0.46–0.68)
None/partial supervision	25	128/506	13.8	4.7–22.8	0.80 (0.71–0.86)
**Completion of treatment**					
≥90%	55	187/1,353	6.0	2.0–9.9	0.78 (0.71–0.83)
<90%	39	89/494	6.5	0.7–12.4	0.50 (0.28–0.66)

Event rate and 95% CI are **in bold** if CIs for two or more strata do not overlap.

a In all but one trial, if therapy was intermittent initially the same schedule was continued throughout therapy. In trials where therapy was daily initially, it was given daily, thrice, or twice weekly thereafter, but outcomes were not different, so these regimens were considered equivalent.

b In a few trials, the number of drugs was the same throughout—these were classified according to the starting regimen.

As shown in Figure S1A, the pooled relapse rate was 10% in the randomized trials of patients with INH resistance, compared to 15% in the subgroup with INH resistance in randomized trials of new cases (Figure S1B), with substantial variation in rates in different studies. As shown in Table 4, relapse rates were significantly higher if rifampin was used for only 2 mo. Nonsignificant but potentially important differences in relapse rates were seen with biweekly dosing schedules in the initial intensive phase, and with fewer effective drugs in the initial intensive phase. As shown in Table 5, acquired drug resistance (i.e., amplification of drug resistance) was significantly lower with use of streptomycin or a greater number of effective drugs in the initial intensive phase. As with failure, acquired drug resistance was progressively less frequent with longer use of pyrazinamide, although all CIs overlapped. Death during treatment was not analyzed as a primary outcome because most studies did not report the timing of death, and early or late deaths during treatment may have very different risk factors. Mortality during treatment was higher in patients treated with regimens for 1 y or longer (unpublished data). After accounting for this, death during treatment was not associated with any other treatment characteristic (unpublished data).

**Table 4:** Stratified analysis of covariates associated with TB treatment outcomes in RCT of patients with INH resistance in new or previously treated cases: Relapse in isoniazid mono-resistance.

Factor	Arms (*N*)	Events/Patients (*N*)	Pooled Event Rate	(95% CI)	I^2^ (95% CI)
**Rifampin use**					
Rifampin 1–2 mo	18	43/196	**23.8**	**11.6–36.0**	**0.44 (0.02–0.68)**
Rifampin 3–5 mo	10	10/83	9.2	0.9–17.5	0 (0–0.60)
Rifampin 6–7 mo	43	46/479	7.1	3.4–10.9	0 (0–0.35)
Rifampin 8+ mo	17	38/409	4.6	0.6–8.6	0.58 (0.29–0.76)
**Frequency of therapy in the initial intensive phase** a					
Daily throughout	60	76/738	9.3	5.2–13.5	0.25 (0–0.47)
Thrice weekly throughout	25	46/353	7.3	1.5–13.1	0.07 (0–0.39)
Twice weekly throughout	3	15/76	13.4	0–33.3	0 (0–0.73)
**Duration of PZA**					
No PZA	25	45/365	10.9	3.6–18.2	0.53 (0.26–0.70)
1–3 mo	38	78/641	10.1	5.1–15.2	0.28 (0–0.53)
4 mo or more	25	14/161	6.4	1.4–11.3	0 (0–0.43)
**Duration of streptomycin**					
No streptomycin	38	77/705	7.5	3.3–11.7	0.41 (0.13–0.60)
1–3 mo	28	46/328	13.2	6.4–20.0	0.43 (0.11–0.64)
4 mo or more	22	14/134	6.9	1.1–12.8	0 (0–0.45)
**Number of drugs to which strains susceptible (effective drugs)**					
***Initial phase*** b					
1 drugs	3	3/17	15.3	0–40.8	0 (0–0.73)
2 drugs	26	24/278	7.7	1.2–14.1	0.11 (0–0.44)
3 drugs	54	105/675	12.2	6.2–18.2	0 (0–0.32)
4 or more drugs	3	5/149	2.6	0–7.9	0 (0–0.73)
***Continuation phase***					
0–1 drugs	43	71/514	9.3	4.0–14.5	0 (0–0.35)
2 drugs	30	55/516	11.1	4.2–17.9	0.52 (0.28–0.69)
3 or more drugs	11	10/83	8.0	0–16.7	0 (0–0.58)
**Supervision of therapy**					
All doses fully supervised	66	107/861	12.3	7.7–16.8	0.36 (0.14–0.53)
None or partial supervision	22	30/306	5.8	1.6–10.0	0 (0–0.46)
**Completion of treatment**					
≥90%	51	97/886	11.1	6.5–15.7	0.40 (0.16–0.57)
<90%	37	40/281	8.2	3.1–13.3	0 (0–0.37)

Event rate and 95% CI are **in bold** if CIs for two or more strata do not overlap.

a In all but one trial, if therapy was intermittent initially the same schedule was continued throughout therapy. In trials where therapy was daily initially, it was given daily, thrice, or twice weekly thereafter, but outcomes were not different, so these regimens were considered equivalent.

b In a few trials, the number of drugs was the same throughout—these were classified according to the starting regimen.

**Table 5:** Stratified analysis of covariates associated with TB treatment outcomes in RCT of patients with INH resistance in new or previously treated cases: Acquired drug resistance in isoniazid mono-resistance.

Factor	Arms (*N*)	Events/Patients (*N*)	Pooled Event Rate	(95% CI)	I^2^ (95% CI)
**Rifampin use**					
Rifampin 1–2 mo	17	6/222	1.9	0–4.3	0 (0–0.50)
Rifampin 3–5 mo	8	2/67	2.0	0–5.8	0 (0–0.65)
Rifampin 6–7 mo	39	51/580	3.6	0.4–6.8	0.33 (0.01–0.55)
Rifampin 8+ mo	9	92/592	7.4	0–15.7	0.91 (0.86–0.95)
**Frequency of therapy in the initial intensive phase** a					
Daily throughout	48	41/739	2.8	0.7–4.9	0 (0–0.41)
Thrice weekly throughout	21	74/496	5.1	0–10.5	0 (0–0.46)
Twice weekly throughout	4	36/226	15.5	0–33.4	0 (0–0.77)
**Duration of PZA**					
No PZA	14	76/473	10.0	1.2–18.8	0 (0–0.71)
1–3 mo	36	71/836	3.7	0.8–6.6	0.55 (0.34–0.69)
4 mo or more	23	4/152	1.2	0–3.1	0 (0–0.45)
**Duration of streptomycin**					
No streptomycin	26	143/963	**12.5**	**7.6**–**17.3**	0.55 (0.30–0.71)
1–3 mo	25	6/347	1.7	0.2–3.3	0 (0–0.43)
4 mo or more	22	2/151	1.1	0–2.8	0 (0–0.45)
**Number of drugs to which strains susceptible (effective drugs)**					
***Initial phase*** b					
0–1 drugs	0	0	—	—	—
2 drugs	21	72/415	**12.0**	**4.0**–**20.0**	0 (0–0.45)
3 drugs	49	79/861	3.8	1.0–6.6	0 (0–0.33)
4 or more drugs	3	0/185	0	0–1.4	0 (0–0.73)
***Continuation phase***					
0–1 drugs	35	56/589	4.5	0.8–8.2	0 (0–0.38)
2 drugs	27	94/790	4.9	0.5–9.2	0.76 (0.65–0.83)
3 or more drugs	9	1/76	0.6	0–2.2	0 (0–0.62)
**Supervision of therapy**					
All doses fully supervised	57	103/1,089	3.1	0.7–5.5	0.51 (0.34–0.64)
None or partial supervision	16	48/372	7.8	0.7–15.0	0.48 (0.07–0.71)
**Completion of treatment**					
≥90%	42	96/1,071	3.4	0.6–6.2	0.6 (0.4–0.8)
<90%	31	55/390	5.0	0.3–9.7	0 (0–0.40)

Event rate and 95% CI are **in bold** if CIs for two or more strata do not overlap.

a In all but one trial, if therapy was intermittent initially the same schedule was continued throughout therapy. In trials where therapy was daily initially, it was given daily, thrice, or twice weekly thereafter, but outcomes were not different, so these regimens were considered equivalent.

b In a few trials, the number of drugs was the same throughout—these were classified according to the starting regimen.

### Meta-regression Results

In meta-regression (Table 6), patients with pretreatment isoniazid resistance had significantly worse treatment outcomes if therapy was intermittent (two or three times weekly) or included fewer effective drugs—in the initial intensive phase. Relapse was lower with longer duration of rifampin or pyrazinamide, but was not associated with duration of post-treatment follow-up. The 95% CI for the dispersion estimates for all three final models included 1, suggesting that the treatment factors included in these models accounted for the majority of the heterogeneity in outcomes seen.

**Table 6:** Adjusted incidence rate ratios of failure, relapse, and acquired drug resistance in INH resistant strains.

Factor	Failure	Relapse	Acquired Drug Resistance^a^
**Duration of rifampin therapy**			
1–2 mo	**4.1 (1.2–13.4)**	**3.8 (1.6–9.0)**	0.7 (0.4–1.3)
3–4 mo	0.8 (0.2–5.0)	1.9 (0.7–5.2)	1.0 (0.3–3.0)
5–7 mo	1.0 (reference)	1.0 (reference)	1.0 (reference)
≥8 mo	1.6 (0.8–2.8)	1.0 (0.5–2.0)	2.1 (0.9–4.9)
Overall significance (*p*-value)^b^	(0.004)	(0.02)	(0.20)
**Number of drugs in regimen to which strains sensitive**			
***Initial intensive phase***			
0 or 1 drug	**6.9 (1.4–33)**	**5.9 (1.0–33)**	No obs.
2 drugs	2.7 (0.7–10.0)	1.9 (0.6–6.7)	**18 (1.4–99)**
3 drugs	1.6 (0.4–6.0)	3.1 (0.9–9.9)	9.6 (0.8–99)
4 or more drugs	1.0 (reference)	1.0 (reference)	1.0 (reference)
Overall significance (*p*-value)^b^	(0.06)	(0.12)	(0.25)
***Continuation phase***			
0 or 1 drug	2.5 (0.7–9.0)	1.0 (0.5–2.3)	1.8 (0.5–6.5)
2 drugs	2.2 (0.6–8.5)	1.2 (0.7–2.5)	1.2 (0.3–5.0)
3 or more drugs	1.0 (reference)	1.0 (reference)	1.0 (reference)
Overall significance (*p*-value)^b^	(0.36)	(0.80)	(0.25)
**Frequency of therapy**			
Initial daily	1.0 (reference)	1.0 (reference)	1.0 (reference)
Thrice weekly throughout	**3.6 (1.6–7.7)**	2.1 (1.0–4.9)	2.1 (0.9–4.5)
Twice weekly throughout	**3.3 (1.8–6.2)**	**5.8 (1.8–18. 2)**	**1.6 (1.1–2.3)**
Overall significance (*p*-value)^b^	(0.0003)	(0.01)	(0.14)
**Streptomycin**			
Not used	1.0 (reference)	1.0 (reference)	1.0 (reference)
Used (2 wk or more)	**0.4 (0.2–0.9)**	1.1 (0.5–2.2)	**0.5 (0.2–1.0)**
Overall significance (*p*-value)^b^	(0.007)	(0.80)	(0.003)
**Pyrazinamide**			
Not used	1.0 (reference)	1.0 (reference)	1.0 (reference)
Used (2 wk or more)	1.2 (0.6–2.0)	**0.5 (0.2–1.0)**	1.5 (0.9–2.3)
Overall significance (*p*-value)^b^	(0.52)	(0.05)	(0.15)

IRR, incidence rate ratios, from negative binomial regression. The percentage completing treatment, mean age, mean percentage male, mean percentage smear positive, and duration of post-treatment follow-up were not significantly associated with any of the three outcomes in preliminary models, so they were dropped from the final model. Incidence rate ratios and 95% CI are **in bold** if significantly different from reference group.

a Acquired drug resistance in failures and/or relapses combined.

b Significance of each factor in multivariate model from log likelihood ratio test.

## Discussion

The most striking finding of this review is the remarkable lack of evidence in support of the WHO recommended retreatment regimen currently used to treat as many as one million TB patients every year [2]. The current body of evidence for treatment of previously treated patients is a dog's breakfast—a few cohort studies in patients with pretreatment drug sensitivity testing, a limited number of randomized trials in previously treated patients, and many small subgroups with initial drug resistance included in trials of new cases. Yet these are the only available results, and so must be extrapolated, albeit very cautiously, to the enormous challenge in resource-limited settings of treating the large number of patients, with a wide spectrum of drug resistance, who require retreatment.

This review is a reminder that, unlike the current initial treatment regimens, the current retreatment regimen was not tested and refined in a sequence of randomized trials. Instead, this regimen was the product of expert opinion [3]. Importantly, this was designed for settings with low prevalence of initial drug resistance and patients who had been treated with a regimen that included rifampin only for the first 2 mo [3]. In this situation, the regimen has high success rates [76]. But treatment outcomes are much worse when used in settings with high initial drug resistance, for patients who previously received regimens with rifampin throughout [2],[6],[77].

The most important limitation of the review is also the most striking finding—the absence of published trials evaluating the current retreatment regimen and the remarkably small number of trials evaluating treatment in previously treated patients. None of these trials included HIV co-infected patients—limiting applicability of the findings to this important population. Because trials compared very different treatment regimens, we could not pool within-trial estimates of treatment effects, which could result in potential confounding. For example, virtually all intermittent therapy was wholly supervised; if intermittent therapy in the initial phase was inferior, this would make supervised therapy appear worse in stratified analysis. The across-trial analysis is also more questionable when outcomes are very heterogeneous. For example, failure and relapse rates differed considerably between the four trials, which evaluated apparently similar regimens of at least 12 mo of rifampin plus ethambutol [30],[31],[34]–[38]. This may reflect the heterogeneity of study populations and settings, as studies were conducted in low-, middle-, and high-income countries over a span of almost 30 years. We attempted to reduce this heterogeneity by performing stratified analysis for major covariates, and by restricting the pooled analysis to patients with confirmed isoniazid mono-resistant pulmonary TB. We also used a more conservative meta-analytic method for pooled estimates and 95% CIs [18]. Concerns over the heterogeneity from the variety of settings and populations are alleviated somewhat by the consistency of results observed in the stratified analysis and the meta-regression. This consistency provides greater confidence in the most important associations found.

A primary objective of this review was to compare the efficacy of different treatment regimens for INH mono-resistance. To accomplish this, we have analyzed the per-protocol results from each trial, using standardized microbiological definitions. All studies reviewed reported adverse events, dropouts, and defaulters separately, facilitating our approach. However, we did not include these outcomes because their definitions and ascertainment were not well standardized, potentially creating greater between-study variability. As well, differences between studies in providers and populations might have had greater influence on these outcomes than the differences in treatment response, potentially creating substantial bias with our analytic approach. Excluding patients who dropped out or defaulted would tend to underestimate treatment effects if these outcomes had been associated with the same treatment characteristics as failure or relapse. However, when this was examined, we found that the proportion that completed therapy was not significantly associated with any of the important treatment characteristics.

We did not study the effect of rifampin dose—largely most studies used the same dose (10–12 mg/kg), limiting power to detected associations of outcomes with dose. However some studies used 450 mg daily, at least initially, which may have been inadequate and contributed to the poor outcomes seen, leaving the optimal dose of rifampin still unresolved. We also could not distinguish between relapse of the same strain of *Mycobacterium tuberculosis* causing the initial infection versus re-infection with a new strain of the bacillus. In settings with high rates of ongoing exposure to *M. tuberculosis*, particularly if HIV seroprevalence is also high, a relatively high proportion of cases of recurrent TB following initial apparent cure may be due to re-infection [78]. However, very few patients had HIV co-infection in the studies reviewed, and in studies with longer follow-up, the great majority of relapses occurred in the first 1–2 y, with very few occurring in the third to fifth years. This suggests re-infection should have accounted for very few of the disease recurrences. Only four studies with 95 patients had less than 1 y follow-up; overall relapse rate in these studies was 10.5%, compared to 11.7% in all studies. Unequal follow-up should not have affected results, since duration of post-treatment follow-up was not associated with relapse or acquired drug resistance in multivariable analysis.

We restricted the research to three languages and three databases, raising the issue of adequacy of our search—germane since we found little evidence and no randomized trials assessing the current WHO regimen. A recent study found that 84% of published papers on TB were published in English, French, and Spanish [79]. Nevertheless, we may have missed some studies because of the language restriction or the database restriction. However, when these findings were presented to expert groups at WHO, the International Union against Tuberculosis and Lung Disease, the Pan American Health Organization, and the American Thoracic Society, no additional relevant studies were identified by those attending. While somewhat reassuring, this cannot be considered systematic, nor evidence.

Given that 10%–20% of all patients receiving TB treatment in low- and middle-income countries require retreatment [2], it is obvious that this regimen plays a key role in the current DOTS strategy. Results with the current WHO retreatment regimen in the cohorts with pretreatment isoniazid resistance or mixed/unknown resistance were disturbingly poor. This may reflect inaccuracies in the performance of the drug sensitivity testing in these earlier studies, although high failure rates [2] and amplification of drug resistance have been documented under programme conditions [4],[77]—supporting our findings. Hence, the most immediate needs identified by this review are to enhance access to accurate drug sensitivity testing for isoniazid and rifampin, and to redesign the recommended standardized retreatment regimen. This redesign should assume that rifampin will have been used throughout initial therapy and follow principles suggested by this review's pooled analysis for isoniazid mono-resistance. Intermittent therapy should be avoided in the initial intensive phase of the re-treatment regimen, at least four effective drugs should be used initially, and at least three effective drugs in the continuation phase. This review also provides support for prolonged use of pyrazinamide and use of streptomycin initially (or an alternative injectable drug in settings with high streptomycin resistance).

The long-term implication of this review is the dire need for evidence-based treatment of patients with drug resistance of all forms—in line with a recent call for research in drug resistant TB [80]. Among new cases, the global weighted mean prevalence of multi-drug resistant TB is 2.9%, but 14.1% have other forms of drug resistance—of whom more than half have isoniazid resistance [81]. The need for trials in MDR TB [82] is inarguable. Although perhaps lacking the cachet of MDR, treatment of these other forms of drug resistance in new cases must also be given priority, for several reasons. First, these other forms of drug resistance are almost five times more common than MDR [81], accounting for 1.3 million new cases in 2008 (extrapolating from the estimated number of 9.2 million new cases [83]. In view of evidence that use of INH preventive therapy may result in increasing levels of INH resistance [84],[85], isoniazid resistance may further increase in countries responding to the “3 I's” initiative of WHO for prevention of HIV-TB [86]. Second, this review has demonstrated the profound weakness of the evidence base for treatment of most forms of drug resistance, including isoniazid resistance. Third, there is good evidence (see [14]) that isoniazid and streptomycin resistance (each alone, or together) result in substantially worse treatment outcomes. Finally, this review and observational studies demonstrate that inadequate re-treatment regimens can result in high rates of failure and relapse with amplified drug resistance. Hence an effective regimen for the group that requires re-treatment could reduce generation of MDR.

A number of priorities for research can be identified from the findings of this review. These include the need for tailoring different regimens for various forms of resistance, the establishment of the minimum number of effective drugs used in the initial or continuation phases (to be balanced against tolerability and costs), the optimal dose of rifampin, the use and timing of intermittent administration, and use and duration of pyrazinamide or an injectable drug (including the utility of streptomycin in settings with high prevalence of streptomycin resistance). There is no doubt that such trials will be challenging, and large numbers of patients will be required. Hence multicenter and multinational randomized trials will be needed. This will require leadership, scientific direction, and funding at an international level [80]. The potential availability of new drugs in the near future increases the need for adequate trials to avoid misuse of these new agents.

## Conclusions

We conclude that there is little published evidence to support the continued use of the currently recommended retreatment regimen. There is an urgent need for a concerted international effort to substantially expand access to reliable drug sensitivity testing and to initiate randomized trials in patients with pretreatment drug resistance of all forms, particularly in previously treated patients. While awaiting results of these trials, the standardized retreatment regimen should be redesigned—at minimum to adequately treat patients with isoniazid resistance. In fact, the upcoming revised treatment guidelines from the WHO will consider these issues and provide some guidance on how to manage previously treated patients [87].

## Supporting Information

Figure S1 Relapse rates. (A) Forest plot of relapse rates in trials of patients only with INH resistance. (B) Forest plot of relapse rates in subgroups with INH resistance in trials of previously untreated patients (new cases).
(9.26 MB TIF)

Table S1 Summary of randomized trials pooled for analysis of isoniazid resistance.
(0.11 MB PDF)

## Abbreviations

DOT: Directly Observed Therapy
DOTS: directly observed treatment short-course
DST: drug susceptibility testing
INH: isoniazid
MDR: multi-drug resistant
RCT: randomized controlled trial
TB: tuberculosis
WHO: World Health Organization
