# Supplementary material for: Standardized Treatment of Active Tuberculosis in Patients with Previous Treatment and/or with Mono-resistance to Isoniazid: A Systematic Review and Meta-analysis
Source: PLoS Med. 2009 Sep 15;6(9):e1000150. doi: 10.1371/journal.pmed.1000150 (PMC2736403; doi:10.1371/journal.pmed.1000150)
Supplement: Table S1 — Summary of randomized trials pooled for analysis of isoniazid resistance. (0.11 MB PDF) [file pmed.1000150.s002.pdf]

Table S1: Summary of randomized trials pooled for analysis of isoniazid resistance

Ref.	Author	Year study began	Country	Total treated (N)	Male (%)	Age of Participants	Outcomes measured Failure	Relapse	ADR
**9 RCT in previously treated cases with isoniazid mono-resistance**									
[1]	HKTBS, BMRC	1970	Hong Kong	205	77	18 and up	Y	N	Y
[2]	Sriyabhaya	1974	Thailand	72	76	18 and up	Y	Y	N
[3–6]	Zierski	1976	Poland	170	NS	NotStated	Y	Y	N
[7]	Babu Swai	1978	India	198	72	18 and up	Y	Y	Y
[8]	Hong	1978	Korea	440	78	18 and up	Y	Y	Y
[9]	Castelo *#	1984	Brazil	13	62	18 and up	Y	Y	Y
[10]	Abdul Aziz *	1986	Pakistan	30	75	NotStated	Y	Y	N
[11]	Narayanan #	1990	India	171	76	12 and up	Y	Y	Y
[12]	ICMR, Chennai *#	1995	India	243	67	12 and up	Y	Y	Y
* Studies where patients with INH mono-resistance were a sub-group of all randomized.									
# Studies included previously treated and untreated patients									
**24 RCT in New cases: subgroups with isoniazid mono-resistance**									
[13]	Figueiredo	1970	Brazil	20	NS	NotStated	Y	Y	N
[14,15]	Singapore/BMRC	1972	Singapore	6	72	NotStated	Y	Y	Y
[16,17]	E Africa/BMRC	1974	E Africa	42	62	18 and up	Y	Y	Y
[18,19]	HK/BMRC	1974	Hong Kong	47	71	18 and up	Y	Y	Y
[20–22]	Singapore/BMRC	1975	Singapore	11	65	18 and up	Y	Y	Y
[23,24]	EA, BMRC	1976	Many	57	67	18 and up	Y	Y	N
[25–27]	HK/BMRC	1977	Hong Kong	40	72	18 and up	Y	Y	Y
[28]	Algeria/BMRC	1977	Algeria	13	44	18 and up	Y	Y	Y
[29]	Mazouni	1977	Algeria	26	NS	NotStated	Y	Y	N
[30,31]	E&C Africa/BMRC	1978	E&C Africa	40	65	18 and up	Y	Y	Y
[32]	Tanzania/BMRC	1978	Tanzania	15	71	18 and up	Y	Y	Y
[33]	HK/Madras BMRC	1978	Hong Kong	7	67	18 and up	Y	Y	Y
[34,35]	Singapore BMRC	1979	Singapore	9	60	18 and up	Y	Y	Y
[36,37]	Zierski	1979	Poland	13	71	18 and up	Y	Y	Y
[38]	Tripathy	1979	India	19	NS	12 and up	Y	N	N
[39]	HK/Madras/BMRC	1980	Hong Kong	12	100	18 and up	Y	Y	Y
[40]	Kenya/Zambia/BMRC	1981	E Africa	42	72	18 and up	Y	Y	Y
[41]	Algeria/BMRC	1981	Algeria	21	65	18 and up	Y	Y	Y
[42]	HK/ BMRC	1983	Hong Kong	59	65	18 and up	Y	Y	Y
[43]	HK/Singapore/BMRC	1983	Singapore	4	66	18 and up	Y	Y	Y
[44,45]	HKCS, BMRC	1984	Hong Kong	6	74	18 and up	Y	Y	Y
[46,47]	TBRC Madras	1985	India	36	70	12 and up	Y	Y	Y
[48]	Agounitestane*	1990	Algeria	13	NS	0 to 80	Y	Y	Y
[29,49]	Chaulet & Mazouni *	1995	Algeria	10	74	18 and up	Y	Y	Y

*These two studies were published in French, all others published in English.
NS: Not stated

Reference List

1. Hong Kong Tuberculosis Treatment Services, Brompton Hospital, British Medical Research Council (1974) A controlled clinical trial of daily and intermittent regimens of rifampicin plus ethambutol in the retreatment of patients with pulmonary tuberculosis in Hong Kong. Tuberc 55: 1-27.

2. Sriyabhaya N, Jittinandana A, Kecharanantana P (1974) Ambulatory intermittent rifampicin and ethambutol in the retreatment of pulmonary tuberculosis. J Med Ass Thailand 57: 550.

3. Zierski M, Bek E, Bergson H, Kucharska A, Szelagowicz B (1976) Retreatment of chronic pulmonary tuberculosis with regimens including high and low doses of rifampicin in the intermittent phase recent and late results - a controlled comparison study. Bull Int Union Tuberc 51: 121-126.

4. National Research Institute for Tuberculosis Poland (1976) A comparative study of daily followed by twice- or once-weekly regimens of ethambutol and rifampicin in the retreatment of patients with pulmonary tuberculosis: second report. Tuberc 57: 105-113.

5. National Research Institute for Tuberculosis Poland (1975) A comparative study of daily followed by twice or once weekly regimens of ethambutol and rifampicin in retreatment of patients with pulmonary tuberculosis. Tuberc 56: 1.

6. Zierski M (1973) A trial of intermittent rifampicin and ethambutol in retreatment regimens. Scand J Resp Dis Suppl. 84: 132-135.

7. Babu SO, Aluoch JA, Githui WA, Thiong'o R, Edwards EA, et al. (1988) Controlled clinical trial of a regimen of two durations for the treatment of isoniazid resistant pulmonary tuberculosis. Tuberc 69: 5-14.

8. Hong YP, Kim SC, Chang SC, Kim SJ, Jin BW, et al. (1988) Comparison of a daily and three intermittent retreatment regimens for pulmonary tuberculosis administered under programme conditions. Tuberc 69: 241-253.

9. Castelo A, Goihman S, Dalboni MA, Jardim J, Kalckman AS, et al. (1989) Comparison of daily and twice-weekly regimens to treat pulmonary tuberculosis. Lancet 334: 1173-1176.

10. Aziz A, Ishaq M, Jaffer NA, Akhwand R, Bhatti AH (1986) Clinical trial of two short-course (6-month) regimens and a standard regimen (12-motnh) chemotherapy in retreatment of pulmonary tuberculosis in Pakistan. Am Rev Resp Dis 134: 1056-1061.

11. Narayanan PR, Tuberculosis Research Centre Chennai (2004) Split-drug regimens for the treatment of patients with sputum smear-positive pulmonary tuberculosis--a unique approach. Trop Med Int Health 9: 551-558.

12. Tuberculosis Research Centre, Indian Council of Medical Research (1997) A controlled clinical trial of oral short-course regimens in the treatment of sputum-positive pulmonary tuberculosis. Int J Tuber Lung Dis 1: 509-517.

13. Poppe de Figeuiredo F, Alves Brito A, Laborne Valle JH, Martins Tavares P, Linhares Trannin P (1974) Short duration chemotherapy of pulmonary tuberculosis: a pilot trial. Bull Int Union Against Tuberculosis 49: 382.

14. Singapore Tuberculosis Service-British Medical Research Council (1977) Controlled trial of intermittent regimens of rifampin plus isoniazid for pulmonary tuberculosis in Singapore. The results up to 30 months. Am Rev Respir Dis 116: 807-820.

15. Singapore Tuberculosis Service, British Medical Research Council (1975) Controlled trial of intermittent regimens of rifampicin plus isoniazid for pulmonary tuberculosis in Singapore. Lancet 306: 1105-1109.

16. Third East African-British Medical Research Councils (1978) Controlled clinical trial of four short-course regimens of chemotherapy for two durations in the treatment of pulmonary tuberculosis: first report. Am Rev Respir Dis 118: 39-48.

17. Third East African-British Medical Research Council (1980) Controlled clinical trial of four short-course regimens of chemotherapy for two durations in the treatment of pulmonary tuberculosis. Second report. Tuberc 61: 59-69.

18. Hong Kong Chest Service and British Medical Research Council (1978) Controlled trial of 6-month and 8-month regimens in the treatment of pulmonary tuberculosis. First report. Am Rev Respir Dis 118: 219-228.

19. Hong Kong Chest Service and British Medical Research Council (1979) Controlled trial of 6-month and 8-month regimens in the treatment of pulmonary tuberculosis: the results up to 24 months. Tuberc 60: 201-210.

20. Singapore Tuberculosis Service-British Medical Research Council (1979) Clinical trial of six-month and four-month regimens of chemotherapy in the treatment of pulmonary tuberculosis. Am Rev Respir Dis 119: 579-585.

21. Singapore Tuberculosis Service-British Medical Research Council (1981) Clinical trial of six-month and four-month regimens of chemotherapy in the treatment of pulmonary tuberculosis: the results up to 30 months. Tuberc 62: 95-102.

22. Singapore Tuberculosis Service-British Medical Research Council (1986) Long-term follow-up of a clinical trial of six-month and four-month regimens of chemotherapy in the treatment of pulmonary tuberculosis. Am Rev Respir Dis 133: 779-783.

23. East African and British Medical Research Councils (1978) Controlled clinical trial of five short-course (4-month) chemotherapy regimens in pulmonary tuberculosis. Lancet 334.

24. East African British Medical Research Councils (1981) Controlled clinical trial of five short-course (4 month) chemotherapy regimens in pulmonary tuberculosis. Am Rev Respir Dis 123: 165-170.

25. Hong Kong Chest-British Medical Research Council (1981) Controlled trial of four twice-weekly regimens and a daily regimen all given for 6 months for pulmonary tuberculosis. Lancet 317: 171-174.

26. Hong Kong Chest Service-British Medical Research Council (1982) Controlled trial of 4 three-times-weekly regimens and a daily regimen all given for 6 months for pulmonary tuberculosis. Second report: the results up to 24 months. Tuberc 63: 89-98.

27. Hong Kong Chest Service-British Medical Research Council (1987) Five-year follow-up of a controlled trial of five 6-month regimens of chemotherapy for pulmonary tuberculosis. Am Rev Respir Dis 136: 1339-1342.

28. Algerian working group-British Medical Research Council (1984) Controlled clinical trial comparing a 6-month and a 12-month regimen in the treatment of pulmonary tuberculosis in the Algerian Sahara. Am Rev Respir Dis 129: 921-928.

29. Mazouni L, Tazir M, Boulahbal F, Chaulet P (1985) Enquête contrôlée comparant trois règimes de chiniothérapie quotidienne de six mois dans la tuberculose pulmonaire, en pratique de routine à Alger. Rev Mal Resp 2: 209-214.

30. East and Central African-British Medical Research Council (1983) Controlled clinical trial of 4 short-couse regimens of chemotherapy (three 6-month and one 8-month) for pulmonary tuberculosis. Tuberc 64: 153-166.

31. East and Central African-British Medical Research Council (1986) Controlled clinical trial of 4 short-course regimens of chemotherapy (three 6-month and one 8-month) for pulmonary tuberculosis: final report. Tuberc 67: 5-15.

32. Tanzania-British Medical Research Council (1985) Controlled clinical trial of two 6-month regimens of chemotherapy in the treatment of pulmonary tuberculosis. Am Rev Respir Dis 131: 727-731.

33. Hong Kong Chest Service-Tuberculosis Research Centre Madras-British Medical Research Council (1989) A controlled trial of 3-month, 4-month, and 6-month regimens of chemotherapy for sputum-smear-negative pulmonary tuberculosis. Results at 5 years. Am Rev Respir Dis 139: 871-876.

34. Singapore Tuberculosis Service-British Medical Research Council (1985) Clinical trial of three 6-month regimens of chemotherapy given intermittently in the continuation phase in the treatment of pulmonary tuberculosis. Am Rev Respir Dis 132: 374-378.

35. Singapore Tuberculosis Service British Medical Research Council (1988) Five-year follow-up of a clinical trial of three 6-month regimens of chemotherapy given intermittently in the continuation phase in the treatment of pulmonary tuberculosis. Am Rev Respir Dis 137: 1147-1150.

36. Zierski M, Bek E, Long MW, Snider DE Jr (1980) Short-course (6 month) cooperative tuberculosis study in Poland: results 18 months after completion of treatment. Am Rev Respir Dis 122: 879-889.

37. Zierski M, Bek E, Long MW, Snider DE Jr (1981) Short-course (6-month) cooperative tuberculosis study in Poland: results 30 months after completion of treatment. Am Rev Respir Dis 124: 249-251.

38. Tripathy SP (1979) Madras study of short-course chemotherapy in pulmonary tuberculosis. Bull Int Union Tuberc 54: 28-30.

39. Hong Kong Chest Service-Tuberculosis Research Centre MBMRC (1991) A controlled clinical comparison of 6 and 8 months of antituberculosis chemotherapy in the treatment of patients with silicotuberculosis in Hong Kong. Am Rev Respir Dis 143: 262-267.

40. Kenyan-Zambian-British Medical Research Council (1989) Controlled clinical trial of levamisole in short-course chemotherapy for pulmonary tuberculosis. Am Rev Respir Dis 140: 990-995.

41. Algerian Working Group British Medical Research Council Cooperative Study (1991) Short-course Chemotherapy for Pulmonary Tuberculosis under Routine Programme Conditions: a comparison of regimens of 28 and 36 weeks duration in Algeria. Tuberc 72: 88-100.

42. Hong Kong Chest Service-British Medical Research Council (1991) Controlled trial of 2, 4, and 6 months of pyrazinamide in 6-month, three-times-weekly regimens for smear-positive pulmonary tuberculosis, including an assessment of a combined preparation of isoniazid, rifampin, and pyrazinamide. Results at 30 months. Am Rev Respir Dis 143: 700-706.

43. Singapore Tuberculosis Service-British Medical Research Council (1991) Assessment of a daily combined preparation of isoniazid, rifampin, and

pyrazinamide in a controlled trial of three 6-month regimens for smear-positive pulmonary tuberculosis. Am Rev Respir Dis 143: 707-712.

44. Hong Kong Chest Service, Tuberculosis Research Centre M, British Medical Research Council (1984) A controlled trial of 2-month, 3-month, and 12-month regimens of chemotherapy for sputum-smear-negative pulmonary tuberculosis. Am Rev Respir Dis 130: 23-28.

45. Hong Kong Chest Service, Tuberculosis Research Centre M, British Medical Research Council (1981) A controlled trial of 2-month, 3-month, and 12-month regimens of chemotherapy for sputum smear-negative pulmonary tuberculosis: the results at 30 months. Am Rev Respir Dis 124: 138-142.

46. Tuberculosis Research Centre Madras (1986) A controlled clinical trial of 3- and 5-month regimens in the treatment of sputum-positive pulmonary tuberculosis in South India. Am Rev Respir Dis 134: 27-33.

47. Balasubramanian R, Sivasubramanian S, Vijayan VK, Ramachandran R, Jawahar MS, et al. (1990) Five year results of a 3-month and two 5-month regimens for the treatment of sputum-positive pulmonary tuberculosis in south India. Tuberc 71: 253-258.

48. Agounitestane D, Chiheb M, Khaled S, Khaled NA, Boulahbal F, et al. (1990) Essai thérapeutique d'une combinaison de trois médicaments essentiels dans la chimiothérapie courte de la tuberculose. Rev Mal Resp 7: 209-213.

49. Chaulet P, Boulahbal F (1995) Essai clinique d'une combinaison en proportions fixes de trois medicaments dans le traitement de la tuberculose. Tuberc Lung Dis 76: 407-412.
